# Supplementary material for: A unique van Hove singularity in kagome superconductor CsV3-xTaxSb5 with enhanced superconductivity
Source: Nat Commun. 2023 Jun 28;14:3819. doi: 10.1038/s41467-023-39500-7 (PMC10300028; doi:10.1038/s41467-023-39500-7)
Supplement: Supplementary file 1 — Supplementary Information [file 41467_2023_39500_MOESM1_ESM.pdf]

Supplementary Materials

A unique van Hove singularity in kagome superconductor $CsV_{3-x}Ta_xSb_5$ with enhanced superconductivity

Yang Luo[1,#], Yulei Han[2,1,#], Jinjin Liu[3,4,#], Hui Chen[5,#], Zihao Huang[5], Linwei Huai[1], Hongyu Li[1], Bingqian Wang[1], Jianchang Shen[1], Shuhan Ding[1], Zeyu Li[1], Shuting Peng[1], Zhiyuan Wei[1], Yu Miao[1], Xiupeng Sun[1], Zhipeng Ou[1], Ziji Xiang[1], Makoto Hashimoto[6], Donghui Lu[6], Yugui Yao[3,4], Haitao Yang[5], Xianhui Chen[1], Hong-Jun Gao[5,*], Zhenhua Qiao[1,*], Zhiwei Wang[3,4,7,*] and Junfeng He[1,*]

[1]Department of Physics and CAS Key Laboratory of Strongly-coupled Quantum Matter Physics, University of Science and Technology of China, Hefei, Anhui 230026, China

[2]Department of Physics, Fuzhou University, Fuzhou, Fujian 350108, China

[3]Centre for Quantum Physics, Key Laboratory of Advanced Optoelectronic Quantum Architecture and Measurement (MOE), School of Physics, Beijing Institute of Technology, Beijing 100081, China

[4]Beijing Key Lab of Nanophotonics and Ultrafine Optoelectronic Systems, Beijing Institute of Technology, Beijing 100081, China

[5]Beijing National Center for Condensed Matter Physics and Institute of Physics, Chinese Academy of Sciences, Beijing 100190, China

[6]Stanford Synchrotron Radiation Lightsource, SLAC National Accelerator Laboratory, Menlo Park, California 94025, USA

[7]Material Science Center, Yangtze Delta Region Academy of Beijing Institute of Technology, Jiaxing, 314011, China

[#]These authors contributed equally to this work.

*To whom correspondence should be addressed:

J.H.(jfhe@ustc.edu.cn), Z.W.(zhiweiwang@bit.edu.cn), Z.Q.(qiao@ustc.edu.cn),

H.-J.G. (hjgao@iphy.ac.cn)

Contents

1. Fermi surface of CsV_3Sb_5 and $CsV_{2.6}Ta_{0.4}Sb_5$ measured at 25K (Fig. S1).
2. Photon energy dependent measurements and location of the momentum cuts along the out-of-plane (K_Z) direction (Fig. S2).
3. First principles calculations on Ta substituted CsV_3Sb_5 (Fig. S3, Fig. S4).
4. Photoemission matrix-element analysis (Fig. S5, Fig. S6).
5. Orbital-resolved band structure calculated for CsV_3Sb_5 and Ta substituted CsV_3Sb_5 (Fig. S7).
6. Comparison of the electron-boson coupling strength in CsV_3Sb_5 and $CsV_{2.6}Ta_{0.4}Sb_5$ (Fig. S8).
7. Gapless states in $CsV_{2.6}Ta_{0.4}Sb_5$ and gapped states in CsV_3Sb_5 near the M point (Fig. S9).
8. Evolution of the van Hove singularity as a function of temperature and Ta substitution (Fig. S10, Fig. S11).
9. A direct link between the enhanced superconductivity and the VHS (Fig. S12, Fig. S13, Fig. S14).
10. The difference of the QPI intensity suppression for V orbitals and Sb orbitals (Fig. S15).
11. The unsymmetrized Fourier transform of the *dI/dV* maps in main Fig. 4 (Fig. S16).
12. Superconducting state of the pristine CsV_3Sb_5 (Fig. S17, Fig. S18).
13. Estimation of the superconducting T_c within the BCS scheme (Fig. S19).
14. Chemical strain effect on superconductivity in CsV_3Sb_5 samples with element substitution (Fig. S20).
15. Calculated total energy profiles for different crystal structures as a function of the Ta substitution level in $CsV_{3-x}Ta_xSb_5$. (Fig. S21).
16. First principles calculations with relaxed/unrelaxed volume of the unit-cell (Fig. S22).

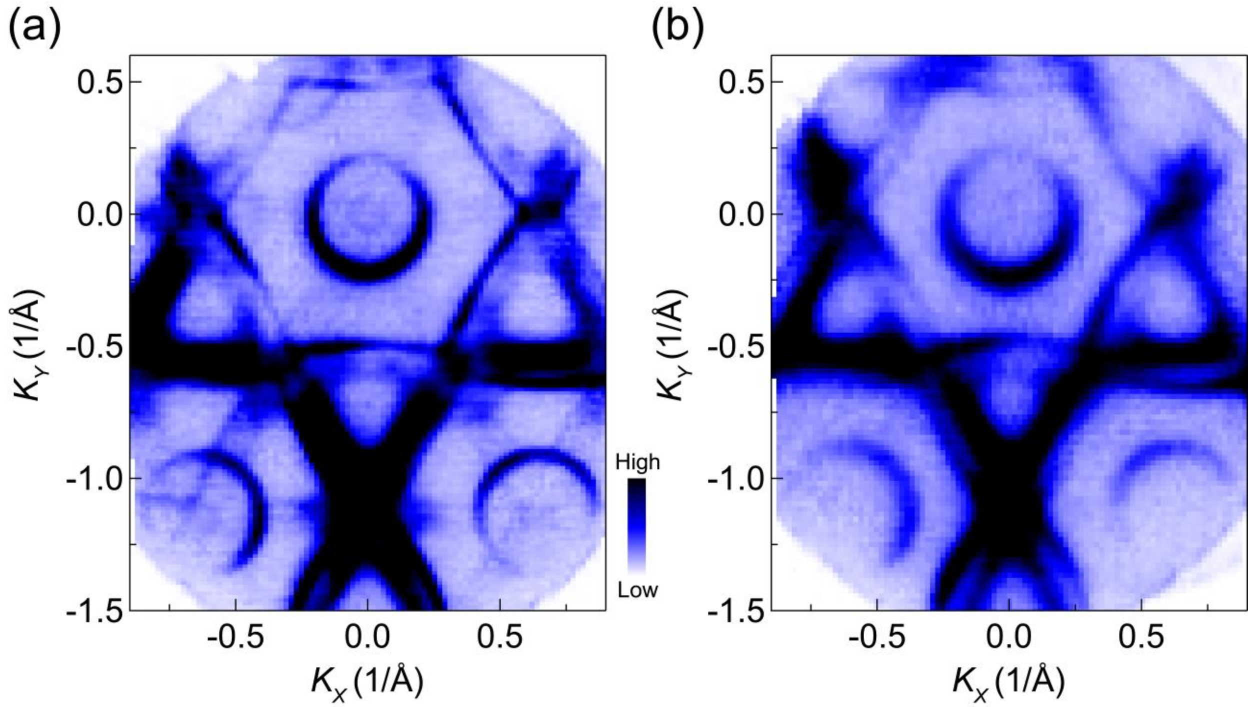

Fig. S1 Fermi surface of CsV_3Sb_5 and $CsV_{2.6}Ta_{0.4}Sb_5$ measured at 25K. (a-b) Fermi surface of CsV_3Sb_5 (a) and $CsV_{2.6}Ta_{0.4}Sb_5$ (b). Same as main Fig. 1(c) and (d), but with an enhanced color scale to highlight the folded Fermi surface sheets in the CsV_3Sb_5 sample.

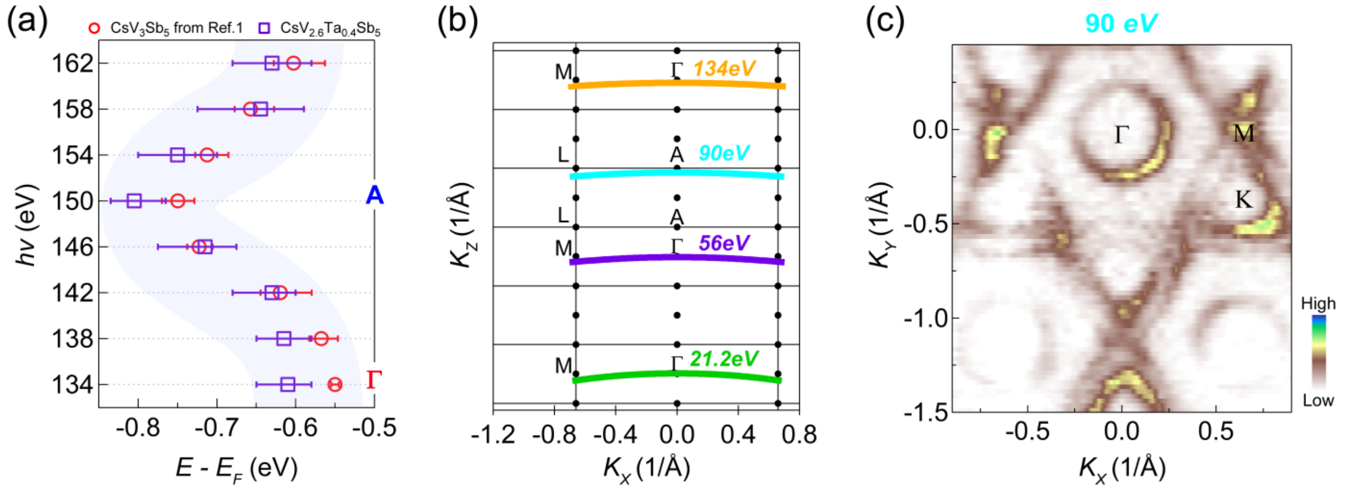

Fig. S2 Photon energy dependent measurements and location of the momentum cuts along the out-of-plane (K_Z) direction. (a) Photon energy dependent measurements have been carried out to study the band evolution along the K_Z direction. The bottom of the electron-like band at the center of the 2D in-plane Brillouin zone is shown as a function of photon energies. Red circles represent the data on CsV_3Sb_5 (from Ref.1) and purple squares represent the data on $CsV_{2.6}Ta_{0.4}Sb_5$. The error bars are from the uncertainties in the determination of the band bottom position. (b) The momentum cuts probed by different photon energies are shown in the K_X-K_Z plane. An inner potential of 11.5 eV is used for the calculation, which has been determined by photon energy dependent measurements. (c) Fermi surface of $CsV_{2.6}Ta_{0.4}Sb_5$ measured at 25K with 90eV photon energy. The overall Fermi surface topology is similar to that in the Γ–K–M plane (Fig. 1d, 56eV), although the Fermi surface in the Γ–K–M plane may exhibit stronger spectral weight around the M point due to the existence of a VHS.

First principles calculations on Ta substituted CsV_3Sb_5

First principles calculations of the band structure have been carried out with one Ta atom and two Ta atoms in 2x2 supercell, respectively (Fig. S3). For the two-Ta-atom substitution, all the four possible structures have been considered (Fig. S4), and the structure with uniformly distributed Ta atoms (structure #4) gives rise to the band structure which is most similar to the experiment. This is also consistent with our sample characterization which shows that the Ta substitution is uniform in the sample. The calculated band structure with one Ta atom per supercell is compared to that of the two Ta atoms with structure #4 (Fig. S3). The overall band structure looks similar, while some slight differences can be identified on the fine features. For example, a small gapped region seems to appear between K and M in the calculation with one Ta atom per supercell.

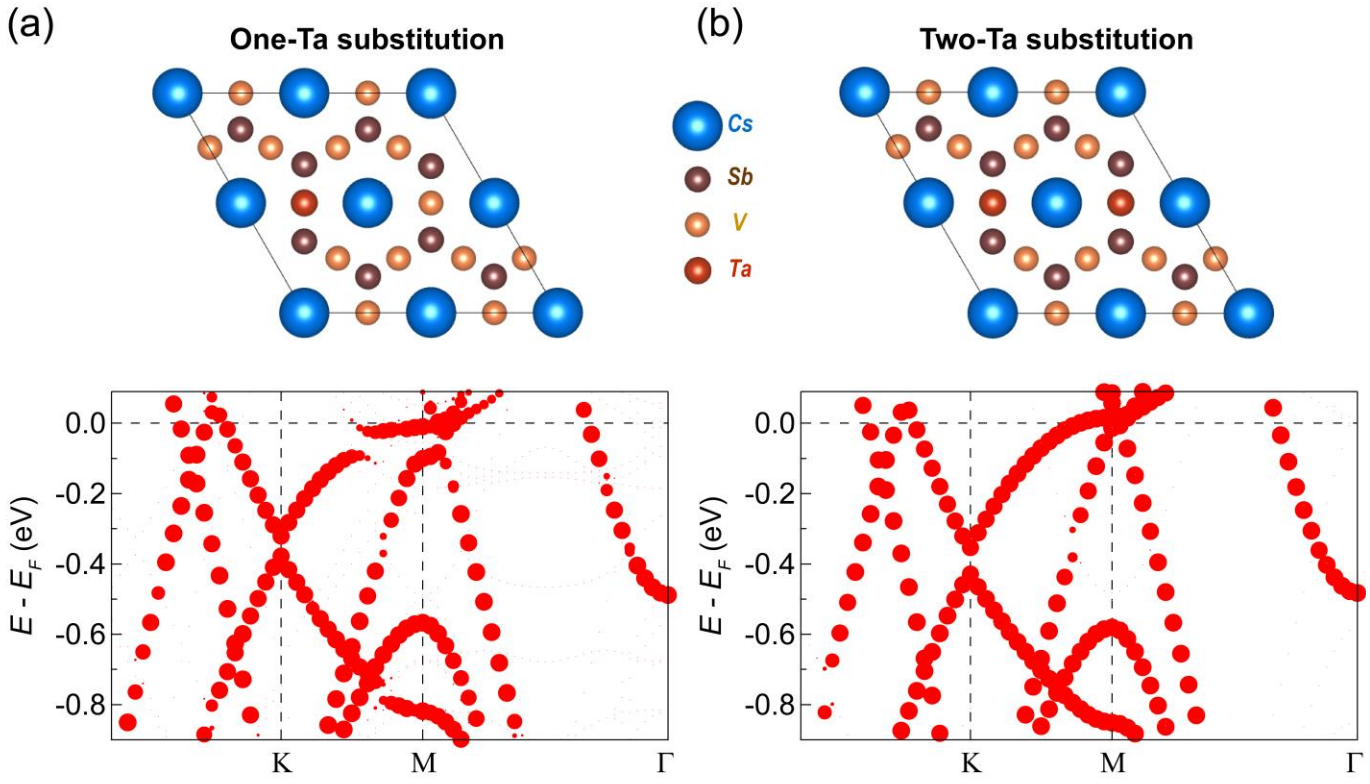

Fig. S3 Calculated band structure with one (two) Ta atom(s) in the 2x2 supercell. (a) The 2x2 supercell structure for one Ta atom substitution, and the calculated band structure along Γ-K-M-Γ. (b) Same as (a) but for two Ta atoms per supercell with structure #4 in supplementary Fig. S4.

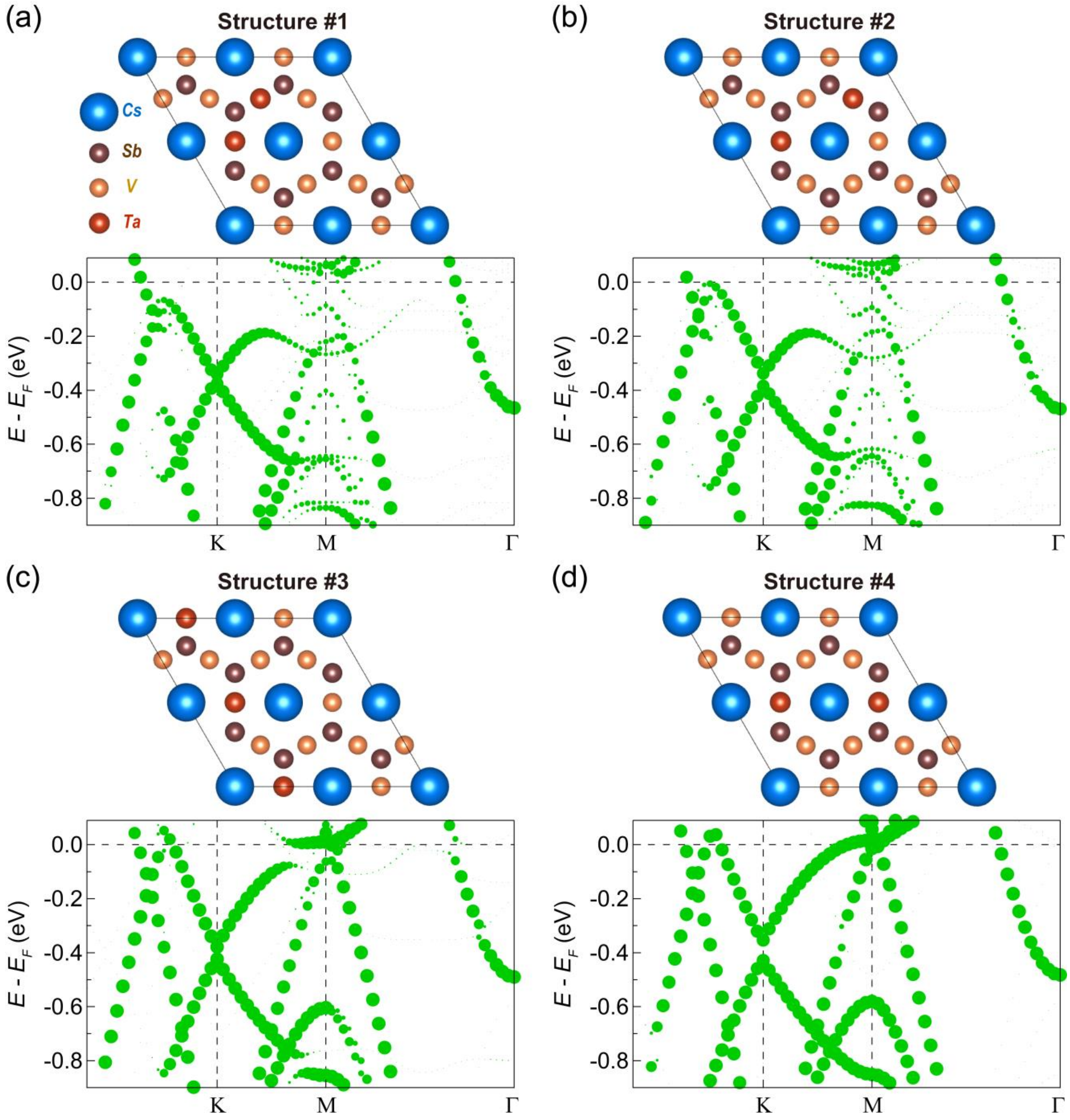

Fig. S4 Calculated band structure with different positions of the two Ta atoms in the 2x2 supercell. (a-d) The four possible structures of two Ta atoms in the 2x2 supercell and the corresponding calculated band structures along the Γ-K-M-Γ direction.

Photoemission matrix-element analysis

In ARPES measurements, different orbitals can be selectively probed by taking advantage of the matrix-element-effect. For instance, if the photoelectron-detector is in the mirror plane of the sample, then orbitals of even (odd) symmetry with respect to the mirror plane can only be detected when the vector potential of the polarized light is in (normal to) the mirror plane. In the following, we present a detailed analysis for the measurements with our experimental setup.

In order to get nonzero photoemission intensity in our measurements, the matrix-element term $\langle \Phi_f^{\mathbf{k}} | \mathbf{A}\cdot\mathbf{p} | \Phi_i^{\mathbf{k}} \rangle$, where $\mathbf{A}$ is the vector potential of the light, must be an even function under the reflection with respect to the mirror plane shown in Fig. S5. The final-state wave function $\Phi_f^{\mathbf{k}}$ has to be even, because odd-parity final state would be zero on the mirror plane. Therefore, a correct combination of the light polarization and the symmetry of the initial state is required to get nonzero photoemission intensity. The general polarization conditions for an overall even matrix-element can be summarized as[2]:

$$\langle \Phi_f^{\mathbf{k}} | \mathbf{A}\cdot\mathbf{p} | \Phi_i^{\mathbf{k}} \rangle \begin{cases} \Phi_i^{\mathbf{k}} \text{ even } \langle + | + | + \rangle \Rightarrow \mathbf{A} \text{ even} \\ \Phi_i^{\mathbf{k}} \text{ odd } \langle + | - | - \rangle \Rightarrow \mathbf{A} \text{ odd.} \end{cases} \tag{1}$$

For our case, we take the d_{xy} orbital measured along the Γ-M direction as an example to elaborate the matrix-element analysis. When the measurement is along the Γ-M direction, the analyzer slit is along the *x* axis (Fig. S5a, c). Therefore, the d_{xy} orbital has an odd-parity with respect to the mirror plane (Fig. S5a, d). To get nonzero photoemission intensity, the LH polarized light is needed, which has an odd component with respect to the mirror plane. Similar analysis can be applied to other orbitals measured along different directions, and the results are summarized in Fig. S6.

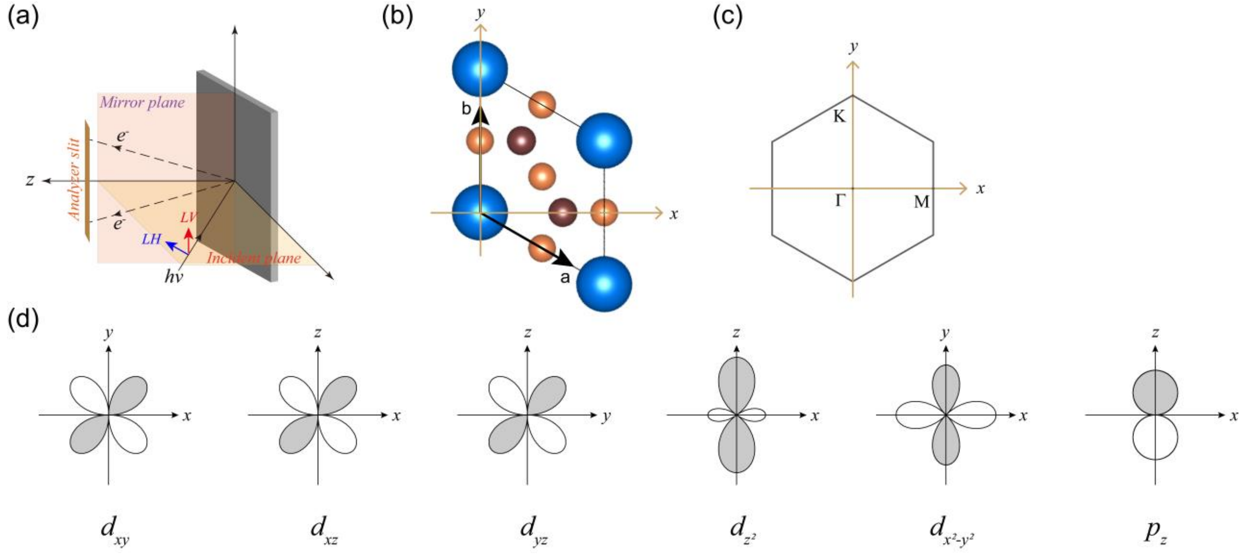

Fig. S5 Experimental geometry and symmetry of different orbitals. (a) Experimental geometry of the ARPES system for our measurements. The analyzer slit is vertical to the floor. (b-c) Schematic of the two-dimensional unit cell in the ab plane and the corresponding two-dimensional Brillouin zone. (d) Schematic of different orbitals.

(a)

High symmetry direction \ Orbitals	d_{xy}	d_{xz}	d_{yz}	d_{z^2}	$d_{x^2-y^2}$	p_z
ΓM	odd	even	odd	even	even	even
ΓK	odd	odd	even	even	even	even

(b)

High symmetry direction	Experimental geometry \ Orbitals	d_{xy}	d_{xz}	d_{yz}	d_{z^2}	$d_{x^2-y^2}$	p_z
ΓM	LV	×	√	×	√	√	√
	LH	√	×	√	×	×	×
ΓK	LV	×	×	√	√	√	√
	LH	√	√	×	×	×	×

Fig. S6 Summary of the photoemission matrix-element analysis. (a) The symmetry analysis of different orbitals measured along Γ-M and Γ-K, respectively. (b) The photoemission matrix-element analysis with different polarizations of the incident light.

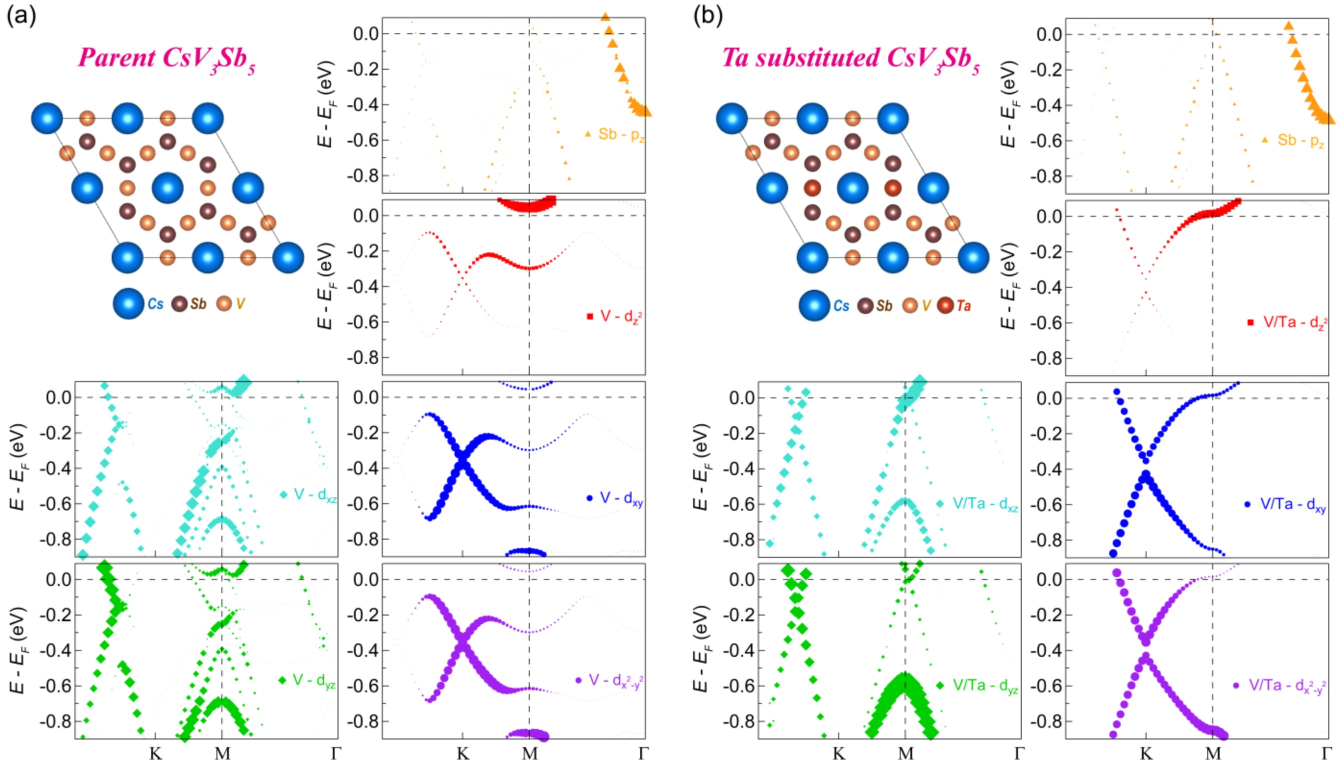

Fig. S7 Orbital-resolved band structure calculated for CsV_3Sb_5 and Ta substituted CsV_3Sb_5. A 2×2×1 "Inverse Star of David" supercell is used to calculate the band structure of CsV_3Sb_5 in the CDW state (a), and a 2×2×1 supercell is constructed with the Ta/V ratio of 1/5 to calculate the band structure of Ta substituted CsV_3Sb_5 (b). The calculated band structures are the same as those in main Fig. 2c, d, but different orbitals are now presented separately. The size of the markers represents the spectral weight of the orbitals.

Comparison of the electron-boson coupling strength in CsV_3Sb_5 and $CsV_{2.6}Ta_{0.4}Sb_5$

In pristine CsV_3Sb_5, electron pairing on the electron-like Fermi pocket around Γ has been suggested. In this context, our observed electron-boson coupling on the same band might serve as a potential pairing glue. While future studies are required to examine the specific nature of this bosonic mode and its potential role in developing the superconductivity in CsV_3Sb_5, it would be useful to compare the electron-boson coupling strength in CsV_3Sb_5 and $CsV_{2.6}Ta_{0.4}Sb_5$.

We estimate the electron-boson coupling strength in CsV_3Sb_5 and $CsV_{2.6}Ta_{0.4}Sb_5$ in two ways.

First, in order to qualitatively determine the relative electron-boson coupling strength in the pristine and Ta substituted samples, we estimate the effective coupling strength λ' following a methodology used in the cuprates[3]. The ratio between the high-energy velocity above the kink energy and the dressed velocity below the kink energy is defined as $\lambda'+1$. As discussed in ref.3, λ' is proportional to the real coupling strength with an overestimation in the quantitative level. Nevertheless, it provides a direct tool to compare the relative coupling strength without any assumption for the data analysis. It is clear that the electron-boson coupling strength in $CsV_{2.6}Ta_{0.4}Sb_5$ is weaker, or at least not stronger, than that in the pristine CsV_3Sb_5 (e.g. compare main Fig. 2h and i).

Second, we can also try to extract the effective Real and Imaginary parts of electron self-energy in both materials. In this process, a bare band dispersion is typically required. Since it is still an open question on how to choose the real bare dispersion of a material, we have explored several methods to extract the effective Real and Imaginary parts of electron self-energy. (1) A parabolic band is used as the bare dispersion, which matches well with the experimental band dispersion in the high energy range. By subtracting the parabolic bare dispersion from the measured dispersion (Fig. S8a), the effective Real part of electron self-energy is obtained (Fig. S8b). (2) The Imaginary part of electron self-energy is extracted from the width of momentum distribution curves (MDCs) by applying the velocity of the bare dispersion (Fig. S8c), and the Real part of electron self-energy can be calculated by the Kramers-Kronig transformation (Fig. S8d). (3) For comparison, the Imaginary part of electron self-energy is also extracted from the MDC width by using the Fermi velocity in the experimental data (Fig. S8e), and the Real part is calculated accordingly (Fig. S8f). All these methods have been used to analyze the data on both the pristine CsV_3Sb_5 (Fig. S8a-f) and the $CsV_{2.6}Ta_{0.4}Sb_5$ compound (Fig. S8g-l). We note that the assumptions for these methods are different. However, regardless of the specific method of data analysis, two key conclusions can be clearly drawn: the energy of the bosonic mode remains similar; the electron-boson coupling strength in $CsV_{2.6}Ta_{0.4}Sb_5$ is not stronger than that in the pristine CsV_3Sb_5.

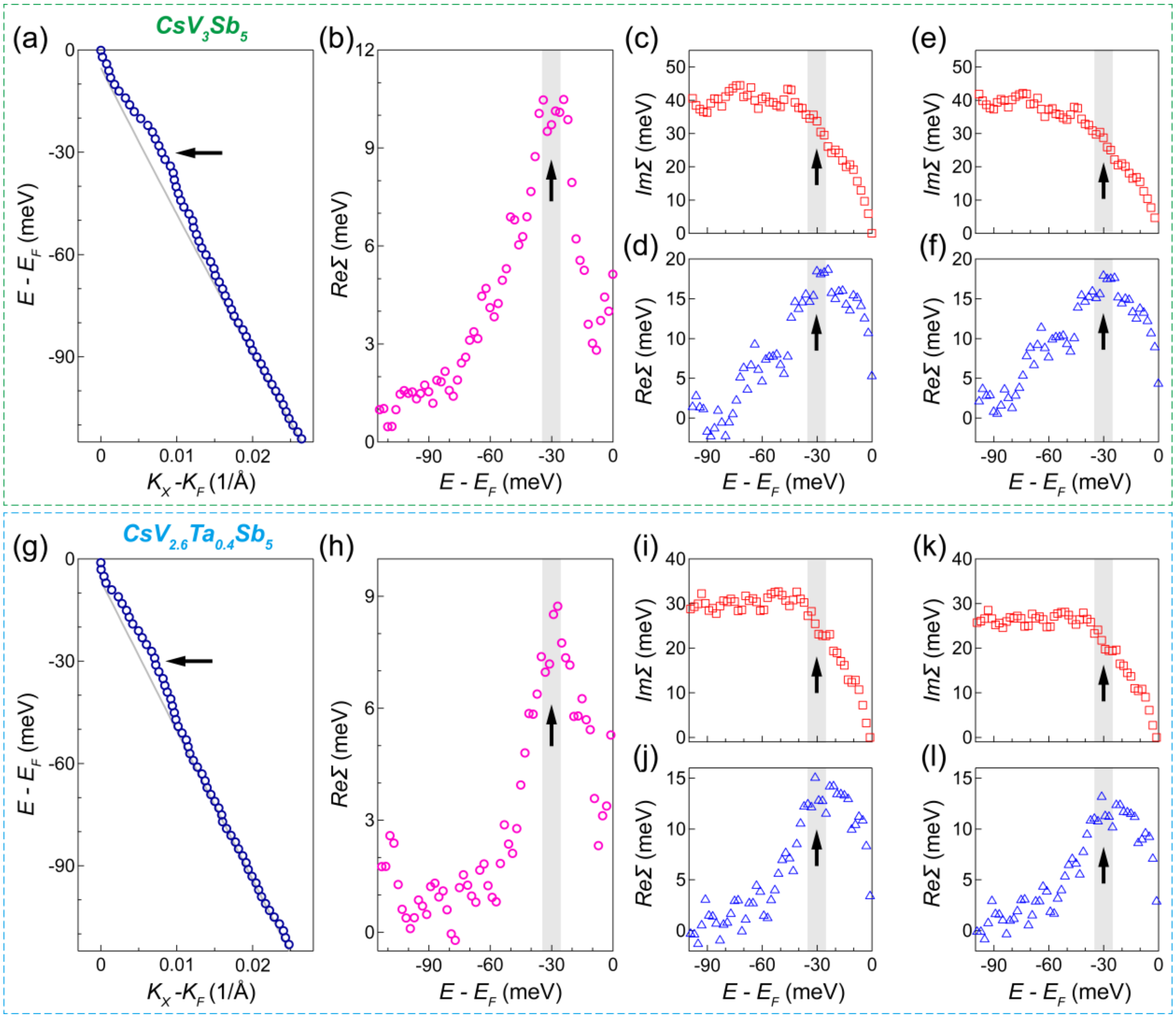

Fig. S8 Comparison of the electron-boson coupling strength in CsV_3Sb_5 and $CsV_{2.6}Ta_{0.4}Sb_5$. (a) MDC-derived dispersion of CsV_3Sb_5. A parabolic bare band is shown in gray. (b) The effective Real part of electron self-energy extracted from (a). (c) The Imaginary part of electron self-energy extracted from the MDC width by using the electron velocity of the bare band. The absolute value at E_F is subtracted to get rid of the contribution from impurity scattering[4]. (d) The Real part of electron self-energy calculated from the Imaginary part in (c) by Kramers-Kronig relations. (e) Same as (c), but the Fermi velocity in the experimental data is used to calculate the Imaginary part of electron self-energy. (f) Same as (d), but calculated from (e). (g-l) Same as (a-f), but for $CsV_{2.6}Ta_{0.4}Sb_5$.

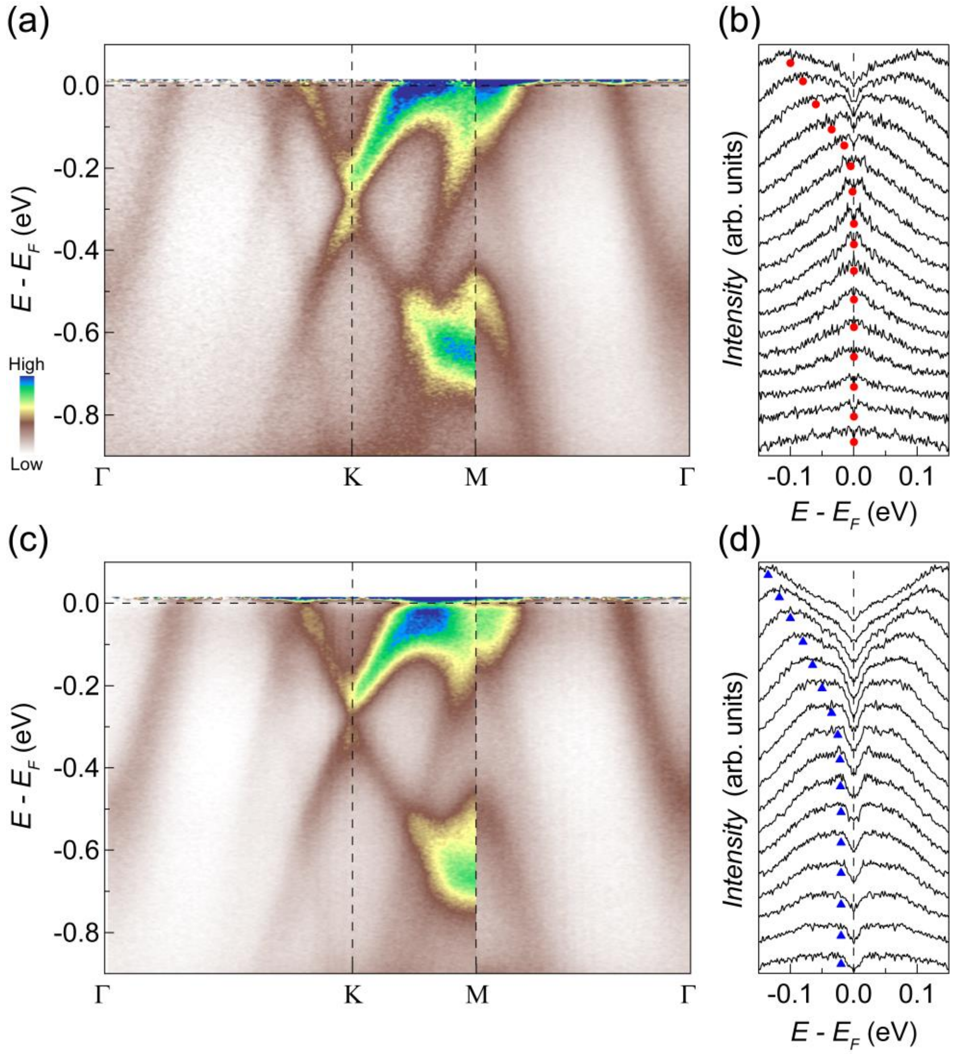

Fig. S9 Gapless states in $CsV_{2.6}Ta_{0.4}Sb_5$ and gapped states in CsV_3Sb_5 near the M point. (a) Photoelectron intensity plots along Γ-K-M-Γ of $CsV_{2.6}Ta_{0.4}Sb_5$ measured at 25K with 21.2eV photons. The Fermi Dirac function has been removed. (b) Symmetrized EDCs near the M point. (c-d) Same as (a-b), but for CsV_3Sb_5. Red circles (blue triangles) mark the EDC peaks measured on $CsV_{2.6}Ta_{0.4}Sb_5$ (CsV_3Sb_5).

Evolution of the van Hove singularity as a function of temperature and Ta substitution

ARPES measurements have been carried out on $CsV_{3-x}Ta_xSb_5$ samples with x=0, 0.1, 0.2, 0.3, and 0.4 (the highest Ta substitution level in this material is ~0.4). As shown in Fig. S10a, the CDW order significantly pushes the VHS away from the E_F. When the CDW is suppressed (either T=200K, or x=0.3, 0.4), the VHS is very close to the E_F. The quantitative examination of the energy distribution curves (EDCs) shows that the VHS in $CsV_{3-x}Ta_xSb_5$ (x=0.3, T_c~4.5K) is slightly above E_F (Fig. S11e), which becomes perfectly aligned with E_F in the samples with x=0.4 (T_c~5.5K, Fig. S11f). This is also consistent with the expectation that Ta substitution could slightly induce electron doping to the system. We note that energy positions of the EDC peaks would be slightly shifted by Fermi-Dirac distribution or second derivative analysis. Therefore, raw EDCs are shown in Fig. S11 with the Fermi-Dirac Function removed.

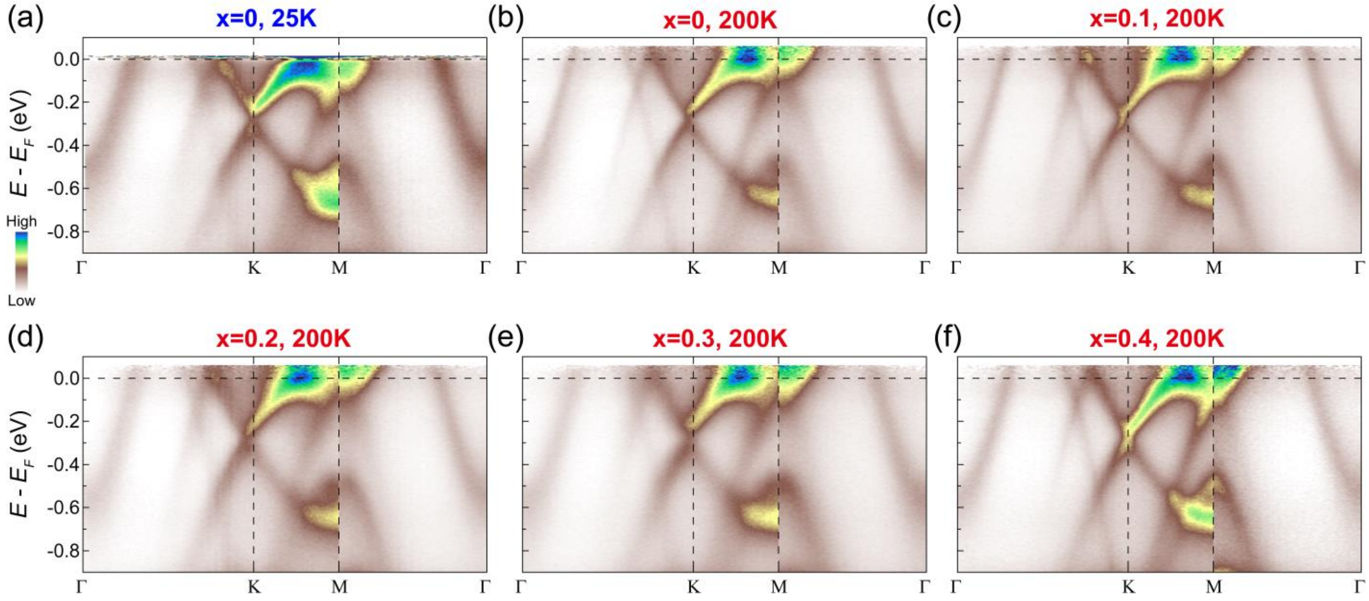

Fig. S10 The energy position of VHS in $CsV_{3-x}Ta_xSb_5$ samples with x=0, 0.1, 0.2, 0.3 and 0.4. (a), (b) Photoelectron intensity plot along Γ-K-M-Γ of the pristine CsV_3Sb_5 sample measured at 25K(a) and 200K(b). (c-f) Photoelectron intensity plots along Γ-K-M-Γ of $CsV_{2.9}Ta_{0.1}Sb_5$ (c), $CsV_{2.8}Ta_{0.2}Sb_5$ (d), $CsV_{2.7}Ta_{0.3}Sb_5$ (e) and $CsV_{2.6}Ta_{0.4}Sb_5$ (f) measured at 200K.

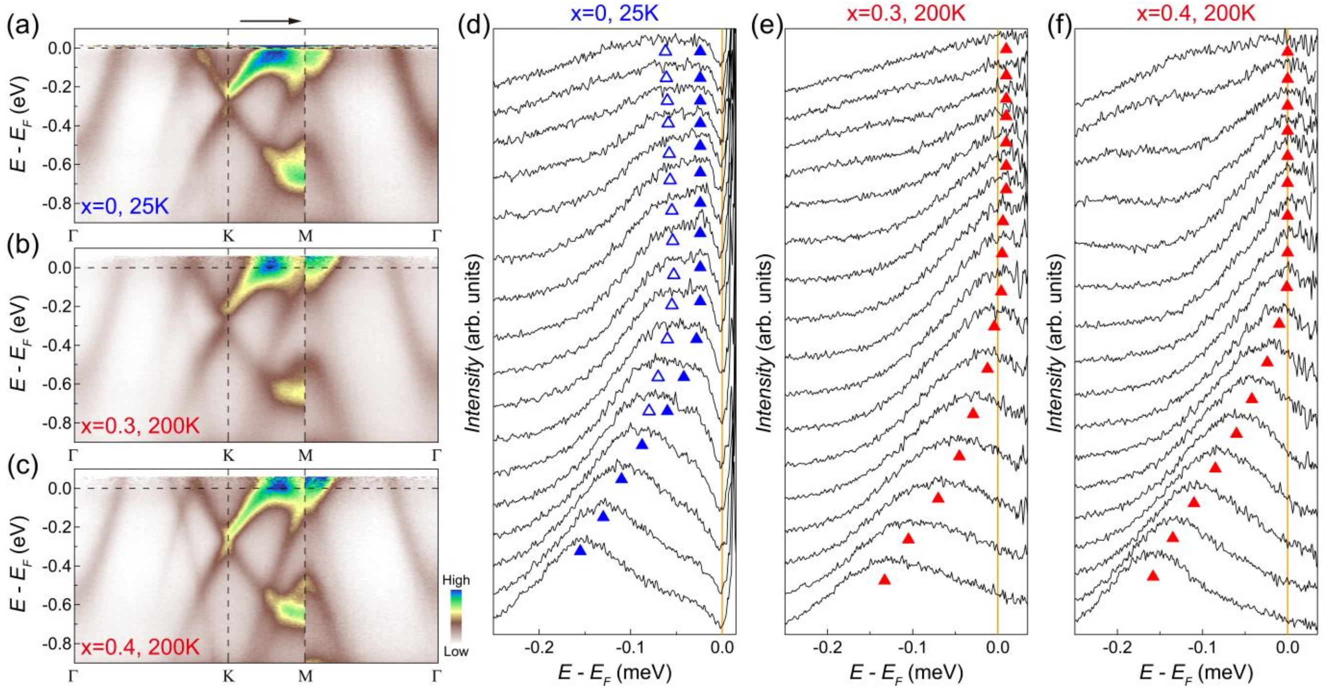

Fig. S11 Quantitative determination of the VHS in $CsV_{3-x}Ta_xSb_5$ samples. (a) Photoelectron intensity plot along Γ-K-M-Γ of the pristine CsV_3Sb_5 sample measured at 25K. (b-c) Photoelectron intensity plot along Γ-K-M-Γ of $CsV_{3-x}Ta_xSb_5$ with x=0.3 (b) and x=0.4 (c) measured at 200K. The Fermi-Dirac function is removed. (d-f) Raw EDCs of (a-c) near the M point. The blue and red triangles in (d-f) indicate the EDC peaks of the VHS.

A direct link between the enhanced superconductivity and the VHS

While a van Hove singularity at the Fermi level would naturally enhance the superconducting T_c, the suppression of competing orders in the Ta substituted sample can also enhance T_c, regardless of the position of VHS. In addition, some competing orders (e. g. CDW order) in the pristine CsV_3Sb_5 primarily involve the correlated V *d*-orbitals. When the competing orders are suppressed in the Ta substituted sample, the possibly enhanced electron correlation of the V *d*-orbitals may also benefit the superconductivity.

In order to avoid the influence from the above factors and establish a direct link between the electron-hole mixed VHS and the substantially enhanced superconducting T_c, a comparison has been made between a Ti substituted sample $CsV_{3-x}Ti_xSb_5$ (x~0.2) and two Ta substituted samples $CsV_{3-x}Ta_xSb_5$ (x~0.3 and x~0.4). In these samples, all the competing orders are completely suppressed by the chemical substitution.

Without the complications from competing orders, we can directly compare the electronic structure and superconductivity of these materials. As shown in Fig. S12, the overall band structure remains similar in all the samples. But a quantitative examination shows that the VHS in the $CsV_{3-x}Ti_xSb_5$ (x~0.2) sample is above E_F, the VHS in the $CsV_{3-x}Ta_xSb_5$ (x~0.3) sample is slightly above E_F, and the VHS in the $CsV_{3-x}Ta_xSb_5$ (x~0.4) sample is perfectly aligned with E_F. These results reveal a clear relation between the energy position of the VHS and the superconducting transition temperature T_c.

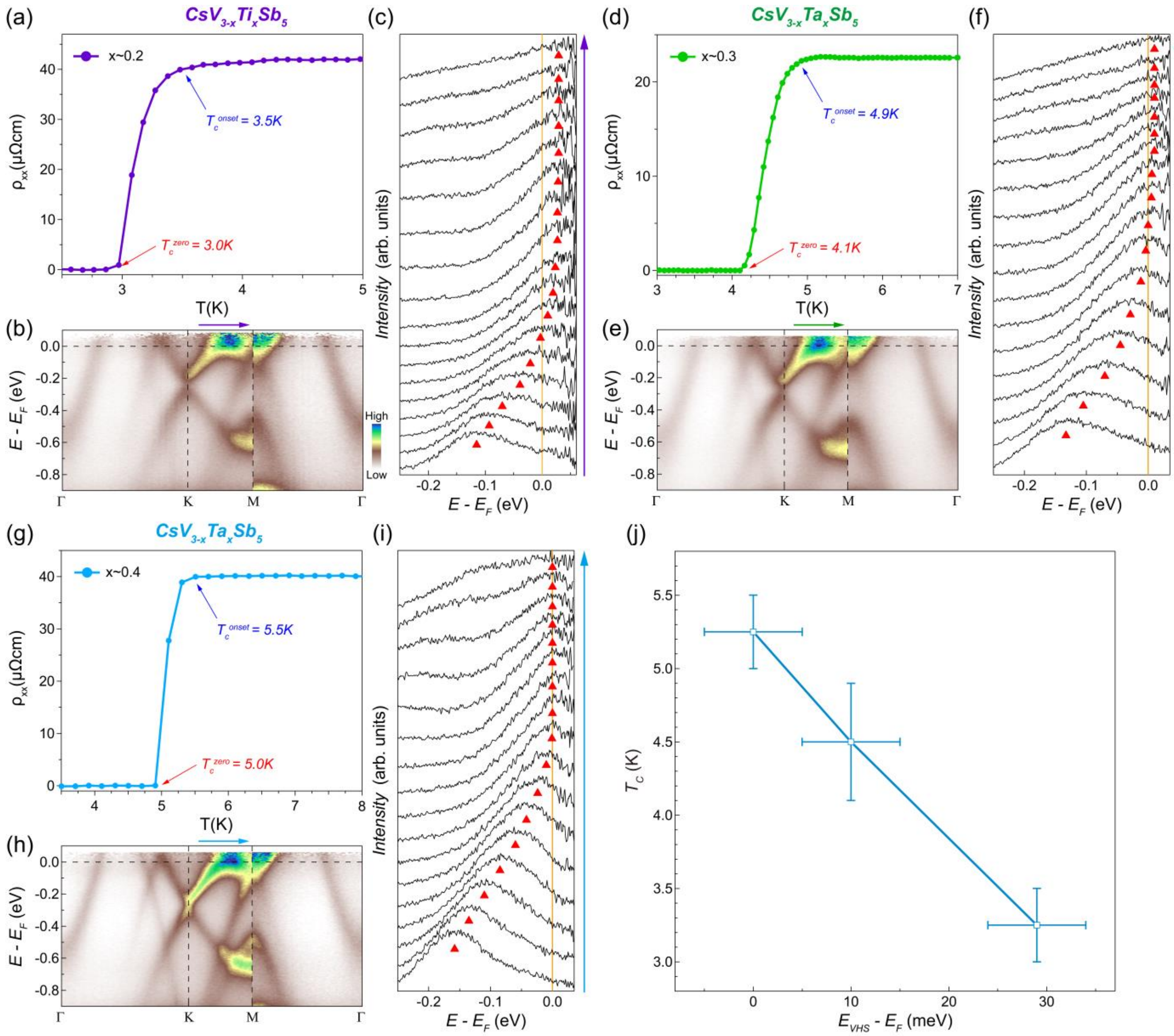

Fig. S12 Superconducting transition temperature T_c and energy position of VHS in $CsV_{3-x}Ti_xSb_5$ and $CsV_{3-x}Ta_xSb_5$ samples. (a) Electrical resistivity as a function of temperature for $CsV_{3-x}Ti_xSb_5$ (x~0.2). (b) Photoelectron intensity plot along Γ-K-M-Γ of the $CsV_{3-x}Ti_xSb_5$ (x~0.2) sample measured at 200K. The Fermi-Dirac function is removed. (c) Raw energy distribution curves (EDCs) of (b) near the M point. (d-f) same as (a-c), but for $CsV_{3-x}Ta_xSb_5$ (x~0.3). (g-i) Same as (a-c) but for $CsV_{3-x}Ta_xSb_5$ (x~0.4). The red triangles in (c), (f) and (i) indicate the EDC peaks of the VHS. (j) Superconducting T_c as a function of the energy position of the VHS. The error bars represent the uncertainties in the determination of the VHS (bottom axis), and superconducting T_c (left axis).

While the above results reveal that the superconducting T_c increases when the VHS moves down towards the Fermi level, it would be interesting to explore whether the superconductivity would be suppressed when the VHS further moves downward to an energy below the Fermi level with even higher electron doping. However, this idea faces a technical challenge, because the Ta substitution limit is x~0.4. In this case, we have carried out *in-situ* surface electron doping on the $CsV_{3-x}Ta_xSb_5$ (x~0.4) sample.

We start with a $CsV_{3-x}Ta_xSb_5$ (x~0.4) sample, where the VHS perfectly locates at the Fermi level (Fig. S13a,c). With *in-situ* Cs doping on the sample surface, the VHS is indeed pushed to an energy slightly below the Fermi level with more Cs adatoms on the sample surface (Fig. S13b,d).

Next, we examine whether the superconductivity would be suppressed with more Cs adatoms on the sample surface. Using low-temperature STM, we have carefully investigated the spatial evolution of the superconducting gap across the boundary between a typical Sb surface with dilute Cs adatoms and a surface area with more Cs adatoms (Fig. S13f). As shown in Fig. S13i-l, while the superconducting gap on the Sb surface shows a typical gap size of the $CsV_{3-x}Ta_xSb_5$ (x~0.4) sample, the superconducting gap decreases on the surface area with more Cs adatoms. This point is also visualized by the 2D map of the superconducting gap $\Delta(\mathbf{r})$ across the boundary (Fig. S13g,h).

The above results reveal that the superconductivity is indeed suppressed while the VHS is further pushed below the Fermi level by surface electron doping beyond the optimal doping (Ta substitution, x~0.4). We note that it is impossible to have exactly the same surface doping in both ARPES and STM measurements, and the ARPES measurements mainly probe an averaged effect of the surface doping. Nevertheless, the qualitative trend is clearly established.

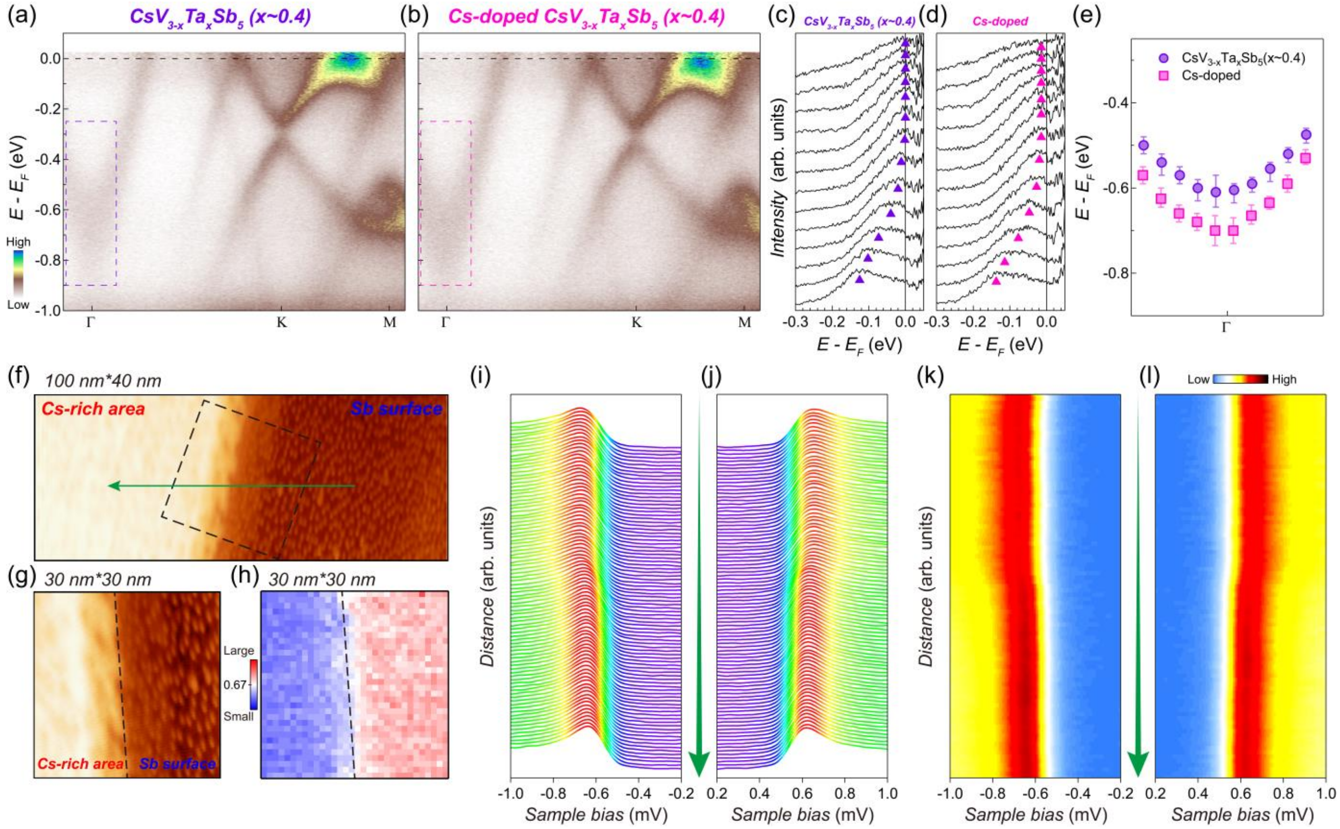

Fig. S13 Suppression of superconductivity by surface electron doping on the CsV$_{3-x}$Ta$_x$Sb$_5$ (x~0.4) sample. (a) Photoelectron intensity plot along Γ-K-M of the CsV$_{3-x}$Ta$_x$Sb$_5$ (x~0.4) sample, measured at 200K. (b) Same as (a), but for the measurement after Cs surface doping on the same sample. (c) EDCs near the VHS in (a). (d) EDCs near the VHS in (b). (e) Electron-like band near Γ before and after Cs surface doping [extracted from the dashed box in (a) and (b)], indicating a charge transfer to the sample by the Cs surface doping. The error bars represent the uncertainties in the determination of the band positions. (f) STM topography near the boundary between a Sb surface area with dilute Cs adatoms (right part) and a surface area with more Cs adatoms (marked as Cs-rich area in the left part). (g) Zoom-in of (f) as labeled by the black dotted square in (f). (h) The 2D map of the superconducting gap $\Delta(\mathbf{r})$ in the same area of (g), measured at 0.05 K. (i-j) Waterfall plot of *dI/dV* spectra along the line-cut across the boundary marked by the green arrow in (f). (k-l) Color plot of the *dI/dV* spectra in (i-j).

After establishing the relationship between the superconductivity and the energy position of the VHS in $CsV_{3-x}Ta_xSb_5$, we can also extend this logic to $CsV_{3-x}Ti_xSb_5$ (x~0.2). In this compound, the VHS locates at around 20-30meV above the Fermi level. So, the VHS should move towards the Fermi level on the surface with more Cs adatoms. Therefore, one should expect a slightly larger superconducting gap on the surface with more Cs adatoms. The change of the gap size should be even less significant than that in the $CsV_{3-x}Ta_xSb_5$ (x~0.4), as the change of VHS has the most significant effect when it is at the Fermi level. However, if this effect can be observed on the $CsV_{3-x}Ti_xSb_5$ (x~0.2), it should show a qualitatively opposite trend comparing to that in the $CsV_{3-x}Ta_xSb_5$ (x~0.4) compound. Therefore, we have carried out similar measurements on the $CsV_{3-x}Ti_xSb_5$ (x~0.2) sample. As shown in Fig. S14, the opposite trend is indeed observed.

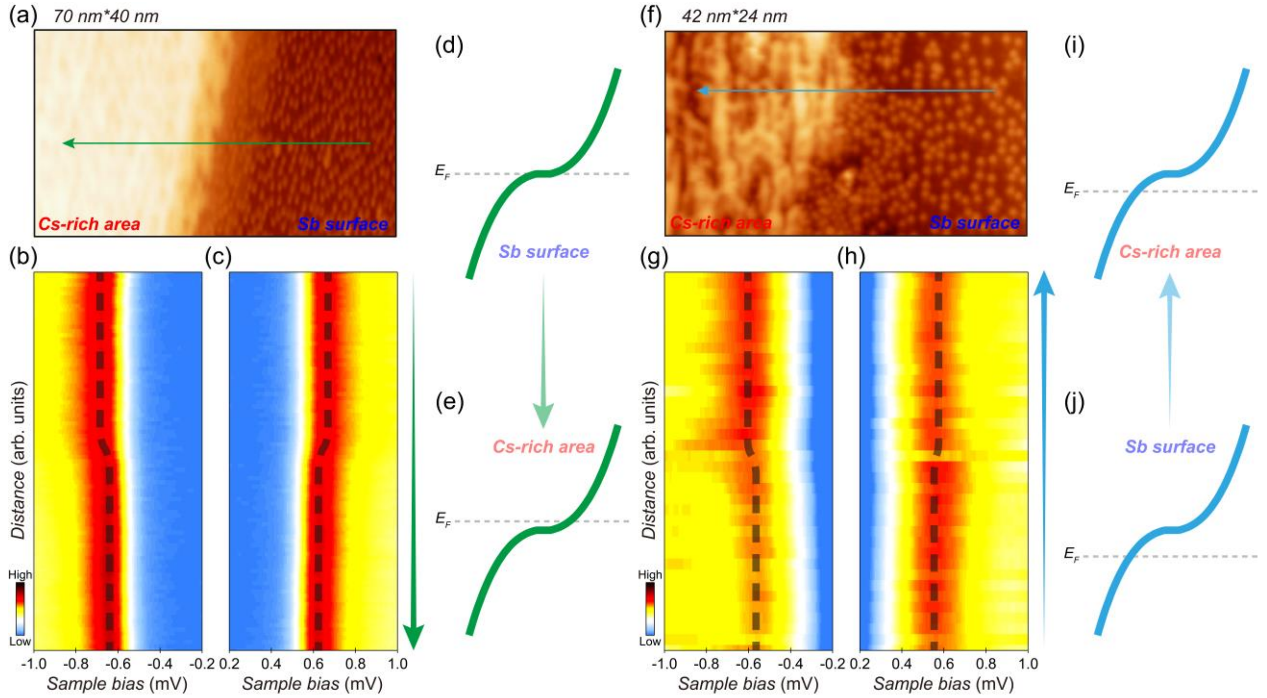

Fig. S14. Opposite surface doping effect on superconductivity in $CsV_{3-x}Ta_xSb_5$ (x~0.4) and $CsV_{3-x}Ti_xSb_5$ (x~0.2). (a) STM topography near the boundary between a Sb surface area with dilute Cs adatoms and a surface area with more Cs adatoms (Cs-rich area), measured on the $CsV_{3-x}Ta_xSb_5$ (x~0.4) sample. (b-c) Color plot of the *dI/dV* spectra measured along the line-cut across the boundary marked by the green arrow in (a). The dashed lines are a guide to the eye. (d-e) Schematic of the evolution of the VHS with surface electron doping. (f-j) Same as (a-e), but for $CsV_{3-x}Ti_xSb_5$ (x~0.2).

These results have clearly established a link between the VHS perfectly aligned with the Fermi level and the record-high T_c in the $CsV_{3-x}Ta_xSb_5$ (x~0.4) compound.

The difference of the QPI intensity suppression for V orbitals and Sb orbitals

In the energy between -0.8meV and -0.4meV, the scattering patterns from the V *d*-orbitals vanish more rapidly than that from the Sb *p*-orbitals (main Fig. 4i-k). In order to perform a quantitative comparison, the QPI intensity in the regions of V orbitals and Sb orbitals are integrated, respectively (Fig. S15). To quantify the difference in the suppression of V orbitals and Sb orbitals, we define

$$\Delta g(E) = \frac{\bar{g}(q_V\,,\ \ E)}{\bar{g}(q_V\,,\ \ -0.9meV)} - \frac{\bar{g}(q_{Sb}\,,\ E)}{\bar{g}(q_{Sb}\,,\ \ -0.9meV)}\ , \tag{2}$$

where $\bar{g}$ refers to the averaged integration of QPI intensity for the V orbitals (q_V) and Sb orbitals (q_{Sb}) at the energy E. The data beyond the superconducting gap (E=-0.9meV) is selected as the reference point.

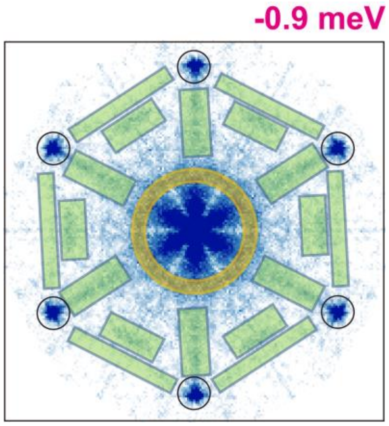

Fig. S15. The QPI intensity integration regions. Six-fold symmetrized Fourier transform of *dI/dV* map measured on the Sb surface of $CsV_{2.6}Ta_{0.4}Sb_5$ at 0.4K, with an energy of -0.9meV. The QPI intensity integration regions of V orbitals and Sb orbitals are marked in green and yellow, respectively.

As shown in main Fig. 4n, the suppression of V orbitals is indeed stronger than that of the Sb orbitals between -0.8meV and -0.4meV. When both orbitals are fully gapped in the zero-conductance region (e.g. between -0.4meV and 0meV), the difference *Δg* becomes zero. We note that *Δg* remains zero at all energies when the superconductivity is suppressed by external magnetic field (main Fig. 4o).

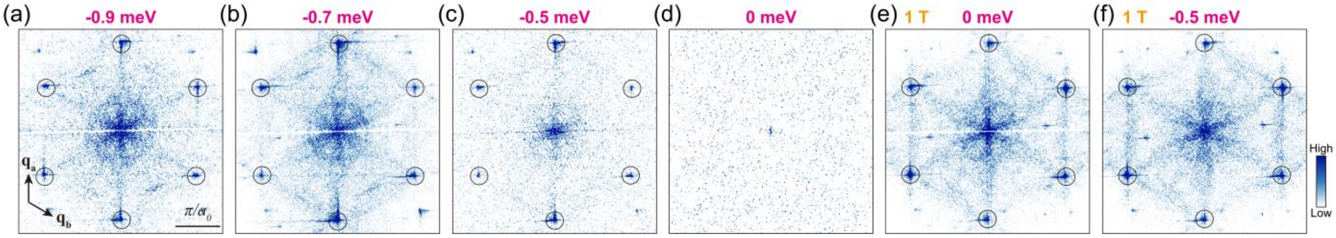

Fig. S16 The unsymmetrized Fourier transform of the *dI/dV* maps in main Fig. 4. (a-d) Unsymmetrized Fourier transform of *dI/dV* maps measured on the Sb surface of $CsV_{2.6}Ta_{0.4}Sb_5$ at 0.4K, with an energy of -0.9meV (a), -0.7meV (b), -0.5meV (c) and 0meV (d), respectively. (e-f) Same as (d) and (c), but measured with a magnetic field of 1T. Black circles indicate the atomic Bragg peaks. STM setup condition: V_{sample}=-1mV, $I_{setpoint}$=1nA, $V_{modulation}$=0.05mV.

Superconducting state of the pristine CsV_3Sb_5

Different from the $CsV_{2.6}Ta_{0.4}Sb_5$ sample, the pristine CsV_3Sb_5 hosts multiple competing orders. In particular, ARPES results demonstrate that most of the V *d*-orbitals are gapped by the CDW order in the pristine CsV_3Sb_5, whereas the Sb *p*-orbitals remain gapless. Therefore, it is naturally expected that the Sb *p*-orbitals can provide electrons to form the Cooper pairs when the material enters the superconducting state from the CDW state. This expectation seems to be consistent with our STM results showing the absence of the scattering pattern from the Sb *p*-orbitals within the superconducting gap (Fig. S17h). However, the systematic energy dependent QPI measurements reveal that the scattering pattern can be clearly identified at the energy around -2meV (Fig. S17d), which becomes less clear at -1meV (Fig. S17e) and disappears at -0.8meV (Fig. S17f). These results indicate that the scattering pattern from the Sb *p*-orbitals starts to disappear beyond the superconducting gap (~0.5meV), which seems to suggest that some other orders should be considered. In order to better understand this phenomenon, we have increased the sample temperature to 4.2K. The superconductivity is killed and the scattering pattern from the Sb *p*-orbitals (the circle at the center) appears again at -0.3meV (Fig. S17i). We note that the circle is as clear as that in Fig. S17d (0.4K, -2meV), and the thermal broadening at 4.2K is insufficient to duplicate the feature at -2meV to -0.3meV. Therefore, it suggests that the energy gap on the Sb *p*-orbitals is closed at 4.2K. Since superconductivity is the only order with a transition temperature (~2.5K) between 0.4K and 4.2K in the pristine CsV_3Sb_5, the temperature dependent results indicate that the Sb *p*-orbitals are indeed involved in the formation of superconductivity, although some other orders may also play a role jointly.

On the other hand, the V *d*-orbitals are primarily involved in the CDW order, but their remnant electron density of states inside the CDW gap may still participate in superconductivity. This idea is not directly supported by the measurement above T_c, in which the scattering patterns from the V *d*-orbitals are not seen at -0.3meV (Fig. S17i). Nevertheless, one cannot rule out this possibility, because the remnant electron density of states of the V *d*-orbitals might not be high enough to be captured by the QPI measurements.

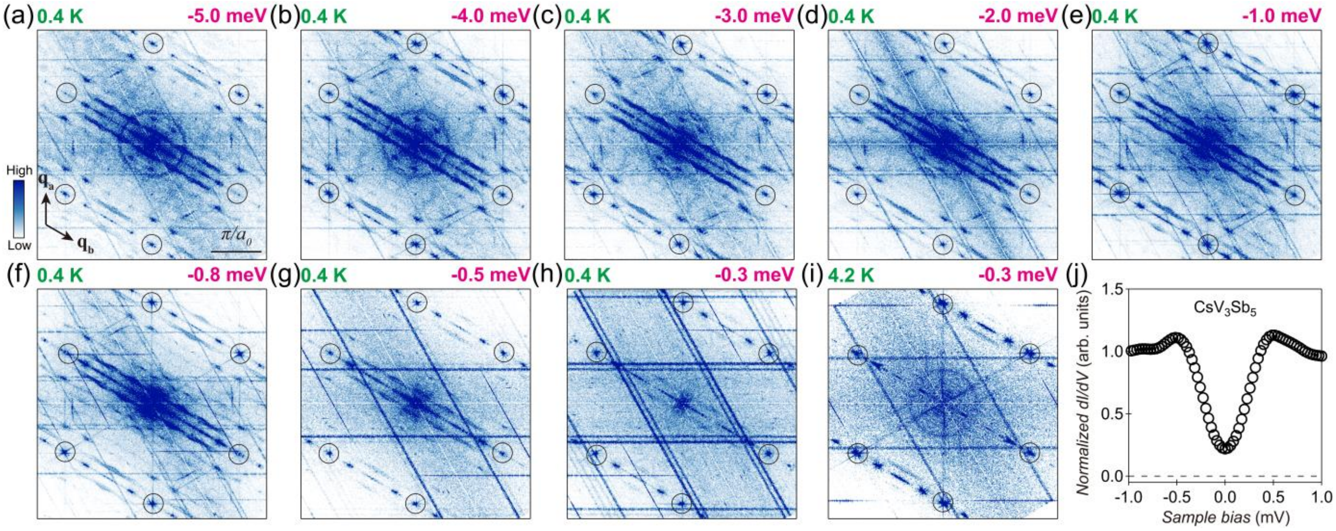

Fig. S17 Fourier transform of *dI/dV* maps in the pristine CsV_3Sb_5. (a-h) Mirror symmetrized Fourier transform of *dI/dV* maps of CsV_3Sb_5 at 0.4K with an energy of -5.0meV (a), -4.0meV (b), -3.0meV (c), -2.0meV (d), -1.0meV (e), -0.8meV (f), -0.5meV (g), and -0.3meV (h), respectively. (i) Mirror symmetrized Fourier transform of the *dI/dV* map at 4.2K with an energy of -0.3meV. (j) The spatially averaged *dI/dV* spectrum at 0.4K. STM setup condition: (a) V_{sample}=-5 mV, $I_{setpoint}$=1 nA, $V_{modulation}$=0.15 mV; (b) V_{sample}=-4 mV, $I_{setpoint}$=1 nA, $V_{modulation}$=0.15 mV; (c) V_{sample}=-3 mV, $I_{setpoint}$=1 nA, $V_{modulation}$=0.1 mV; (d) V_{sample}=-2 mV, $I_{setpoint}$=1 nA, $V_{modulation}$=0.1 mV; (e-g) V_{sample}=-1 mV, $I_{setpoint}$=1 nA, $V_{modulation}$=0.03 mV; (h,i) V_{sample}=-0.3 mV, $I_{setpoint}$=0.1 nA, $V_{modulation}$=0.1 mV.

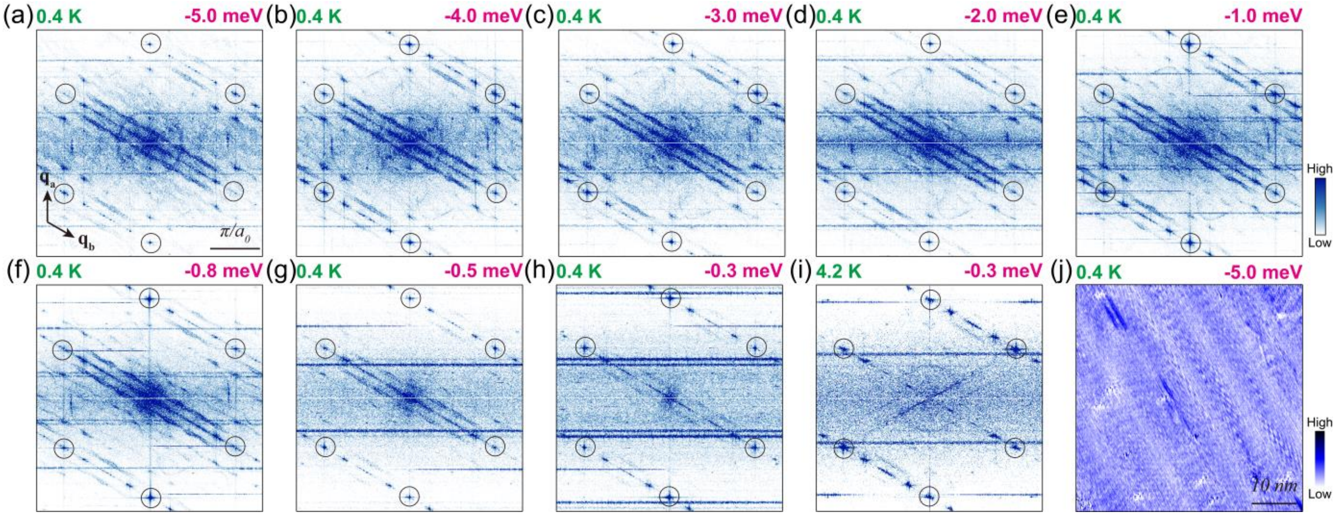

Fig. S18 The unsymmetrized raw data for Fig. S17. (a-h) Unsymmetrized Fourier transform of *dI/dV* maps of CsV_3Sb_5 at 0.4K with an energy of -5.0meV (a), -4.0meV (b), -3.0meV (c), -2.0meV (d), -1.0meV (e), -0.8meV (f), -0.5meV (g), and -0.3meV (h), respectively. (i) Unsymmetrized Fourier transform of the *dI/dV* map at 4.2K with an energy of -0.3meV. (j) The real space *dI/dV* map at 0.4K with an energy of -5.0meV.

Estimation of the superconducting T_c within the BCS scheme

It was argued that the simple electron-phonon coupling is insufficient to account for the T_c of ~2.5K in the CsV_3Sb_5 (ref. 5). Therefore, it would be interesting to see whether the T_c of ~5.5K in $CsV_{2.6}Ta_{0.4}Sb_5$ can be reproduced by the similar model but with enhanced density of states by the VHS.

In order to simplify the estimation, we consider two bands in the system, an electron-like parabolic band centered at the Γ point and a band with the VHS at the M point (Fig. S19a). We estimate T_c by using the BCS formula:

$$1 = V \int_{-k_B\theta_D}^{k_B\theta_D} \frac{N(\varepsilon)}{2\varepsilon} tanh\left(\frac{\varepsilon}{2k_B T_c}\right) \quad (3)$$

where the density of states $N(\varepsilon)$ includes both the VHS band and the electron-like parabolic band. The Debye temperature $\theta_D = 142\text{K}$ and $V \approx \lambda/N_F = 0.048\text{eV}$ are adopted from Ref. 5, which are assumed to be the same for both bands for simplicity. The density of states of the VHS band is estimated by the tight-binding model[6]. In order to visualize the relative enhancement of T_c when the VHS is slightly away from the E_F, we calculate the T_c as a function of the energy difference between the VHS and E_F by moving the VHS band downward continuously (Fig. S19b). T_0 represents the highest T_c we have obtained, when the VHS is exactly at E_F. The absolute value of the calculated T_0 is 0.28 K.

In this sense, it would be interesting to explore whether other bosonic pairing mechanism may exist in this system, especially when broken time-reversal symmetry is suggested by recent experiments.

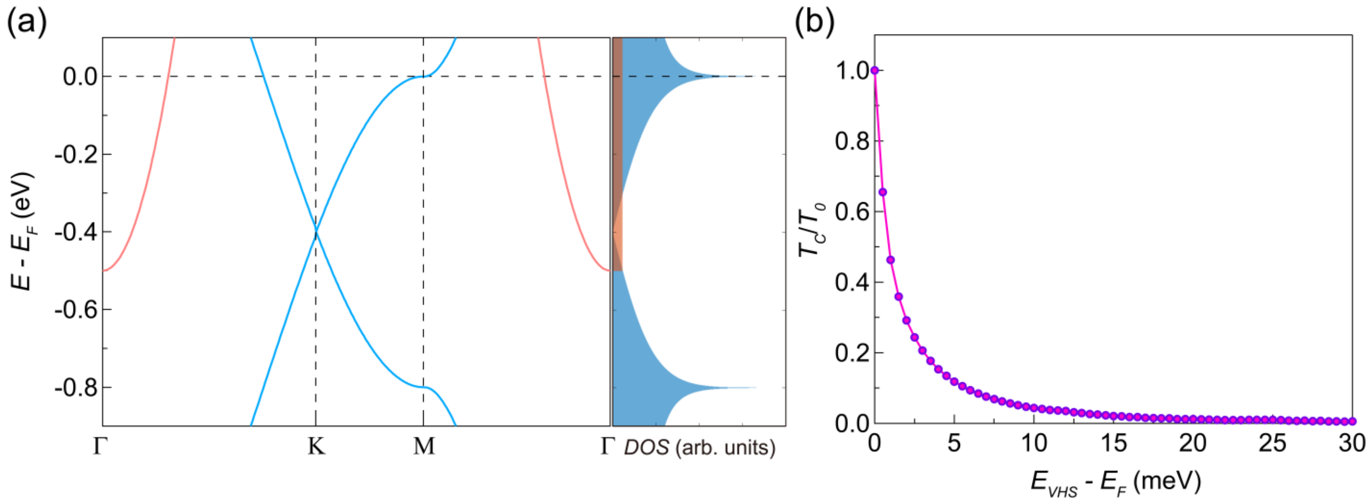

Fig. S19 Estimation of the superconducting T_c within the BCS scheme. (a) Schematic of the band structure, including an electron-like band near Γ and a band with the VHS near M. (b) Calculated T_c as a function of the energy difference between the VHS and E_F.

Chemical strain effect on superconductivity in CsV_3Sb_5 samples with element substitution

In principle, the substitution of V by Ta might induce a chemical strain in the sample. Nevertheless, we find that the substantially enhance T_c is special for the Ta substitution, which is not a universal property in the CsV_3Sb_5 system with the similar chemical strain.

Similar to Ta, the Mo atom is also larger than the V atom, and the substitution of V by Mo would also induce a chemical strain in the sample. We have performed systematic Mo substitution till the highest substitution level that can be successfully grown ($CsV_{3-x}Mo_xSb_5$, x=0.15), and we have observed a suppression of the superconductivity. This is distinct from the Ta substituted samples, where the T_c is enhanced monotonically as a function of Ta substitution till the highest achievable substitution level.

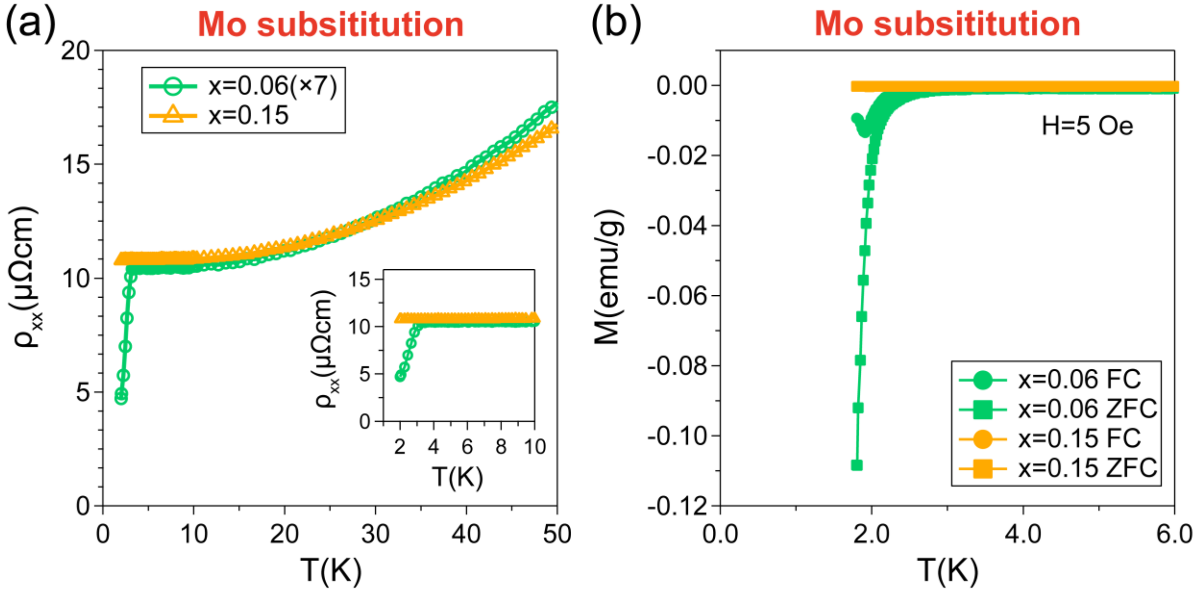

Fig. S20 The superconducting transition temperature of Mo substituted CsV_3Sb_5. (a) Electrical resistivity as function of temperature for $CsV_{3-x}Mo_xSb_5$ samples with x~0.06 and 0.15. We multiply data of x~0.06 by seven for comparison. The data near T_c is shown in the inset with an expanded scale. (b) Field-Cooled (FC) and Zero-field-Cooled (ZFC) magnetization measurement of the Mo substituted samples with H = 5Oe.

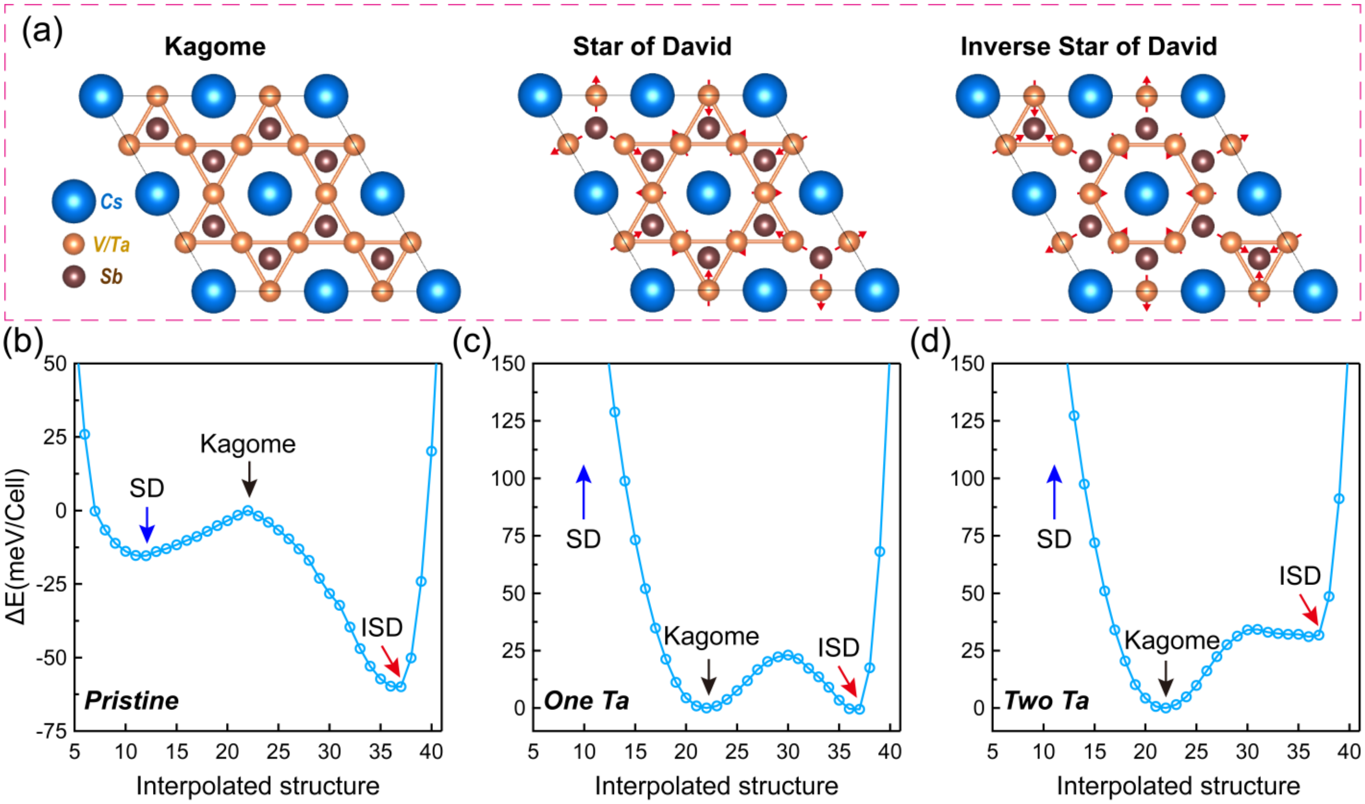

Fig. S21 Total energy profiles for different crystal structures as a function of the Ta substitution level in $CsV_{3-x}Ta_xSb_5$. (a) The 2×2 supercells for kagome structure, the Star of David 2×2 CDW phase, and the Inverse Star of David 2×2 CDW phase. The red arrows indicate the lattice distortion due to the breathing mode. (b-d) Total energy as a function of interpolated structure in pristine CsV_3Sb_5 (b), one Ta atom substitution in the 2×2 supercell (c) and two Ta atoms substitution in the 2×2 supercell (d). The ΔE stands for the relative total energy with respect to the kagome structure per supercell.

First principles calculations with relaxed/unrelaxed volume of the unit-cell

First principles calculations have been carried out with the volume of the unit-cell relaxed and unrelaxed, respectively. While some subtle changes can be found, for example, the size of the small gap at K point, the overall band structure and the position of the VHS are the same.

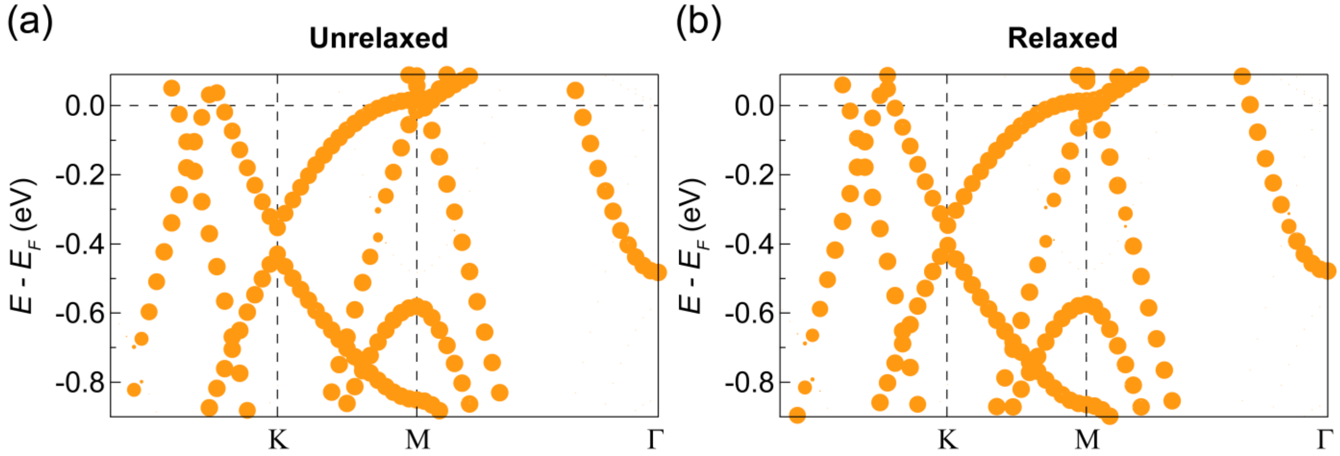

Fig. S22 The calculated band structure of Ta substituted CsV_3Sb_5 samples. (a-b) Band structure along Γ-K-M-Γ calculated with the volume of the unit-cell unrelaxed (a) and relaxed (b). Two Ta atoms are considered in a 2×2 supercell. The positions of Ta atoms are the same as those in supplementary Fig. S4d.

Supplementary References:

1. Luo, Y. et al., Electronic states dressed by an out-of-plane supermodulation in the quasi-two-dimensional kagome superconductor CsV_3Sb_5. *Phys. Rev. B* **105**, L241111 (2022).
2. Damascelli, A., Hussain, Z. & Shen, Z.-X. Angle-resolved photoemission studies of the cuprate superconductors. *Rev. Mod. Phys*. **75**, 473-541 (2003).
3. Lanzara, A. et al., Evidence for ubiquitous strong electron–phonon coupling in high-temperature superconductors. *Nature* **412,** 510-514 (2001).
4. Juan, J. et al., Many-body interactions and Rashba splitting of the surface state on Cu(110). *Phys. Rev. B* **89**, 085404 (2014).
5. Tan, H. et al. Charge Density Waves and Electronic Properties of Superconducting Kagome Metals. *Phys. Rev. Lett.* **127**, 046401 (2021).
6. Mizoguchi, T. & Udagawa, M. Flat-band engineering in tight-binding models: Beyond the nearest-neighbor hopping. *Phys. Rev. B* **99**, 235118 (2019).
